# Universal epitaxy of non-centrosymmetric two-dimensional single-crystal metal dichalcogenides

**DOI:** 10.1038/s41467-023-36286-6

**Published:** 2023-02-03

**Authors:** Peiming Zheng, Wenya Wei, Zhihua Liang, Biao Qin, Jinpeng Tian, Jinhuan Wang, Ruixi Qiao, Yunlong Ren, Junting Chen, Chen Huang, Xu Zhou, Guangyu Zhang, Zhilie Tang, Dapeng Yu, Feng Ding, Kaihui Liu, Xiaozhi Xu

**Affiliations:** 1 Guangdong Provincial Key Laboratory of Quantum Engineering and Quantum Materials, School of Physics and Telecommunication Engineering, South China Normal University, Guangzhou, 510631 China; 2 Guangdong-Hong Kong Joint Laboratory of Quantum Matter, School of Physics and Telecommunication Engineering, South China Normal University, Guangzhou, 510631 China; 3 State Key Laboratory for Mesoscopic Physics, Frontiers Science Center for Nano-optoelectronics, School of Physics, Peking University, Beijing, 100871 China; 4 Key Laboratory for Nanoscale Physics and Devices, Institute of Physics, Chinese Academy of Sciences, Beijing, 100190 China; 5 International Centre for Quantum Materials, Collaborative Innovation Centre of Quantum Matter, Peking University, Beijing, 100871 China; 6 Songshan Lake Materials Laboratory, Institute of Physics, Chinese Academy of Sciences, Dongguan, 523808 China; 7 Shenzhen Institute for Quantum Science and Engineering, Southern University of Science and Technology, Shenzhen, 518055 China; 8 Faculty of Materials Science and Engineering/Institute of Technology for Carbon Neutrality, Shenzhen Institute of Advanced Technology, Chinese Academy of Sciences, Shenzhen, 518055 China

**Keywords:** Two-dimensional materials, Two-dimensional materials

## Abstract

The great challenge for the growth of non-centrosymmetric 2D single crystals is to break the equivalence of antiparallel grains. Even though this pursuit has been partially achieved in boron nitride and transition metal dichalcogenides (TMDs) growth, the key factors that determine the epitaxy of non-centrosymmetric 2D single crystals are still unclear. Here we report a universal methodology for the epitaxy of non-centrosymmetric 2D metal dichalcogenides enabled by accurate time sequence control of the simultaneous formation of grain nuclei and substrate steps. With this methodology, we have demonstrated the epitaxy of unidirectionally aligned MoS_2_ grains on a, c, m, n, r and v plane Al_2_O_3_ as well as MgO and TiO_2_ substrates. This approach is also applicable to many TMDs, such as WS_2_, NbS_2_, MoSe_2_, WSe_2_ and NbSe_2_. This study reveals a robust mechanism for the growth of various 2D single crystals and thus paves the way for their potential applications.

## Abstract (web summary)

Recent studies have reported the growth of 2D non-centrosymmetric single crystals on substrates with surface steps, but the mechanisms are still unclear. Here, the authors demonstrate a method to grow unidirectionally aligned transition metal dichalcogenide grains on various types of substrates, showing the importance of the simultaneous formation of grain nuclei and substrate steps.

## Introduction

The direct synthesis of two-dimensional (2D) single crystals on desired substrates is essential for high-end applications, such as integrated electronic and optoelectronic devices. Among the ~1800 2D materials predicted by high-throughput computation, more than 99.5% have a non-centrosymmetric crystalline structure. During the growth of non-centrosymmetric 2D materials, antiparallel grains are frequently observed because of their energetic equivalency on most substrates^1–14^. To break this equivalence, atomic steps on the surface were introduced and proved effective for the epitaxial growth of single-crystal hexagonal boron nitride (h-BN) and transition metal dichalcogenides (TMDs) on a few specially-designed substrates, such as vicinal Cu(110) and a- or c-plane sapphire. Until now, these successes were attributed to two different mechanisms, (i) step-edge-guided epitaxy, namely the edge-docking mechanism, and (ii) epitaxy guided by both the terraces and the step edges of the substrate, namely the dual-coupling mechanism^15–27^. However, numerous experimental observations showed that, in many cases, the atomic steps cannot guide epitaxial growth, even on the same kind of substrates. Currently, the epitaxy of non-centrosymmetric 2D materials can be achieved only in a very narrow experimental window. The decisive mechanism that ensures the epitaxy of non-centrosymmetric 2D materials remains elusive and is eagerly waiting to be fully explored.

Here, we revealed that the accurate time sequence control of the simultaneous formation of grain nuclei and substrate steps is the key in the growth of single-crystal TMDs. Theoretical calculations reveal that the immature steps on the substrate promote grain nucleation near the step edges and guide the unidirectional alignment of 2D nuclei regardless of the step orientations. With this technique, unidirectionally aligned MoS_2_ grains were achieved on sapphire (a, c, m, n, r and v planes with various step directions), MgO and rutile-TiO_2_ substrates. This epitaxy was also demonstrated applicable to other TMDs like WS_2_, NbS_2_, MoSe_2_, WSe_2_ and NbSe_2_. Our results reveal a robust mechanism for the universal growth of non-centrosymmetric 2D single crystals and thus would enhance the high-end applications of these 2D single crystals.

## Results

### Growth of unidirectionally aligned MoS_2_ grains

**Fig. 1:**
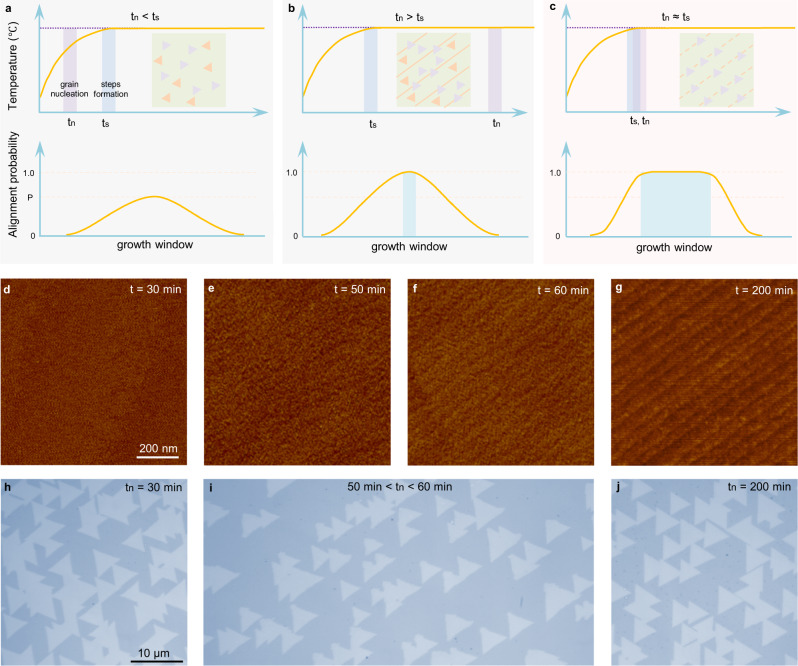
Growth of unidirectionally aligned MoS_2_ grains by the simultaneous-formation-guided mechanism. **a**–**c** Schematic diagrams of the growth behaviour of non-centrosymmetric 2D grains when t_n_ < t_s_ (**a**), t_n_ > t_s_ (**b**) and t_n_ ≈ t_s_ (**c**). T_n_ and t_s_ are the 2D grains nucleation and atomic steps formation time, respectively. The shaded coloured areas represent the time window of t_n_ and t_s_. The violet horizontal dashed lines represent the growth temperature. The yellow horizontal dashed lines correspond to the alignment probability of 1.0 and a certain value P. The growth window of 2D single crystals is greatly broadened when t_n_≈ t_s_. **d**–**g** Atomic force microscopy (AFM) images of vicinal c-Al_2_O_3_ surfaces annealed for different times (t_s_). The steps are invisible for the original substrate (**d**) and are clear after long time annealing (**g**). **e**, **f** The pattern of step edges starts to appear at 50–60 min. **h**–**j** Optical images of MoS_2_ grains on c-Al_2_O_3_ at different nucleation times (t_n_). Unidirectionally aligned MoS_2_ grains were obtained when t_n ≈_ t_s_ (**i**). Otherwise, antiparallel grains were produced (**h, j**). The scale bars in (**d**–**g**) and (**h**–**j**) are the same.

In this study, we show that the time sequence of 2D grain nucleation (t_n_) and atomic step formation (t_s_) on a substrate is the key factor that determines the success of epitaxy. Based on the sequence of t_n_ and t_s_, the behaviour of 2D material growth is distinctly different: (i) if t_n_ < t_s_ or the nucleation of 2D grains occurs before the formation of the step edges, the growth is mainly controlled by the coupling between 2D grains and the substrate and the degeneracy of two antiparallel directions is not broken due to the lack of step edges (Fig. 1a); (ii) if t_n_ > t_s_ or the nucleation of 2D grains occurs after the parallel step edges are formed, one must control the growth condition very carefully to ensure that all the grains nucleate at the step edges, and the epitaxial growth window is generally very narrow (Fig. 1b, such as special cutting angle, special steps direction and extremely high processing accuracy, and see Supplementary Tables 1–3 for details); (iii) t_n_ ≈ t_s_ or the nucleation of 2D grains occurs during the formation process of step edges, these immature step edges are active sites for the nucleation of 2D grains and the unidirectional alignment of the 2D grains is guaranteed in a broad growth window (Fig. 1c, such as high tolerance of substrates lattice structure, steps directions, types of substrate, and suitability of various TMDs growth, and see Supplementary Table 3 for details).

Figure 1d–g shows the step edge formation process on a vicinal Al_2_O_3_ substrate. The pristine vicinal surface is rough and no clear pattern of step edges can be seen, which implies that both terraces and step edges are not well constructed (Fig. 1d). During the annealing process, the pattern of step edges can be seen at ~50–60 min (Fig. 1e, f), but the complete formation of parallel straight step edges requires additional ~150 min of annealing (Fig. 1g). Thus, the time of the atomic step formation (t_s_) can be determined to be 50–60 min under this annealing condition. Experimentally, the time of MoS_2_ nucleation (t_n_) can be controlled by the time of feeding the reactor with sulphur flux. We found that the epitaxy growth of unidirectionally aligned MoS_2_ grains can be easily realized when t_n_ ≈ t_s_, or by feeding the sulphur flux to the reactor during the step edge formation process (Fig. 1i and Supplementary Fig. 1). In contrast, feeding the sulphur either too early or too late (i.e., t_n_ < t_s_ or t_n_ > t_s_) always leads to a poor alignment of MoS_2_ grains (Fig. 1h, j).

Systematic structural characterizations confirmed that once the unidirectionally aligned grains were realized, the parallel grains would seamlessly stitch into high-quality single-crystal films, consistent with all previous reports on graphene, hBN and TMDs^26–28^. The three-fold rotational symmetry of the low-energy electron diffraction (LEED) pattern (Supplementary Fig. 2) and the identical orientations of the polarized second-harmonic generation (SHG) pattern (Supplementary Fig. 3) confirmed the unidirectional alignment of MoS_2_ grains grown on the vicinal c-plane sapphire (c-Al_2_O_3_) surface and their seamless coalescences. The absence of any dark lines in the SHG mapping (Supplementary Fig. 4a, b) or optical image of H_2_O-etched films (Supplementary Fig. 4c, d) and the perfect crystalline lattices shown in the scanning transmission electron microscopic (STEM) images (Supplementary Fig. 5) demonstrated the single crystallinity of the MoS_2_ films.

Optical and electrical measurements also reveal that the as-grown MoS_2_ films are of high quality. The circular helicity in the polarized photoluminescence (PL) spectrum is as high as 80% (Supplementary Fig. 6a, b), which is competitive with exfoliated single-crystal MoS_2_ flakes^29^. The full width at half maximum (FWHM) of the excitation peak is approximately 50 meV in the PL spectrum at room temperature (Supplementary Fig. 6c), and the representative peak difference of *A*_1g_ and *E*_2g_ is ~19 cm^−1^ in the Raman spectrum (Supplementary Fig. 6d), both suggesting that the samples are high-quality monolayer MoS_2_. We also fabricated field-effect transistor (FET) devices at different locations of single-crystal MoS_2_ on 300 nm SiO_2_/Si substrates. The highest mobility reaches ~45 cm^2^ (Vs)^−1^ and the average mobility is ~38 cm^2^ (Vs)^−1^ measured at room temperature (Supplementary Figs. 7, 8), which is also comparable to that of exfoliated ones^30^.

### Growth of MoS_2_ on c-Al_2_O_3_ with different step directions

**Fig. 2:**
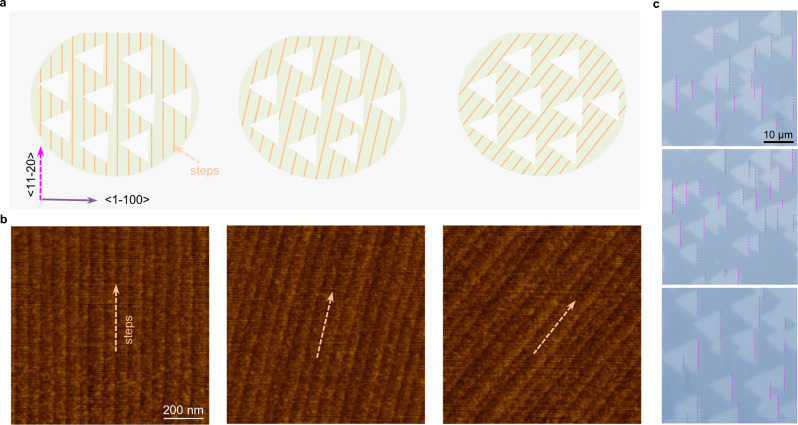
Growth of unidirectionally aligned MoS_2_ grains on vicinal c-Al_2_O_3_ with different step directions. **a** Schematic diagrams of MoS_2_ grains on the surface with different step directions. The orange lines correspond to the parallel steps. **b** AFM images of the c-Al_2_O_3_ surface. Different substrates exhibited different step directions. **c** Optical images of MoS_2_ grains on Al_2_O_3_ substrates shown in (**b**). One edge of all MoS_2_ grains is along the <11–20> direction of Al_2_O_3_ (calibrated by the cutting edge of the Al_2_O_3_ wafer). The dashed fuchsia lines show parallel edges of MoS_2_ grains. The scale bars in (**b**) and (**c**) are the same.

In previous growth of non-centrosymmetric 2D single crystals, such as h-BN and TMDs, the epitaxy requires atomic steps along a certain direction of the substrate and can only be realized on specially-designed substrates after tremendous experimental attempts^18–27^. Astonishingly, we observed that the unidirectional alignment of MoS_2_ grains grown in our experiments can be realized on various vicinal Al_2_O_3_ substrates regardless of the step orientations. We custom-fabricated vicinal c-Al_2_O_3_ substrates with various cutting directions (Fig. 2a) that determined the alignments of the step edges (Fig. 2b). Among the 9 kinds of step directions we tested, all the grown MoS_2_ grains were aligned along the <11–20> direction of the c-Al_2_O_3_ surface (Fig. 2c and Supplementary Fig. 9). This result confirms the advantage of the previously proposed dual-coupling mechanism^27^, where the 2D materials epitaxy is guided by both the terraces and the step edges of the substrate: (i) the sapphire terrace-MoS_2_ interaction leads to two preferred antiparallel orientations of the MoS_2_ crystal and (ii) the sapphire step edge-MoS_2_ interaction breaks the symmetry of the antiparallel orientations. This study further shows that the symmetry of the antiparallel aligned MoS_2_ grains can be broken by step edges along different directions, which enables the epitaxial growth of various 2D materials on different substrates.

### Universal growth of MoS_2_ on various substrates

**Fig. 3:**
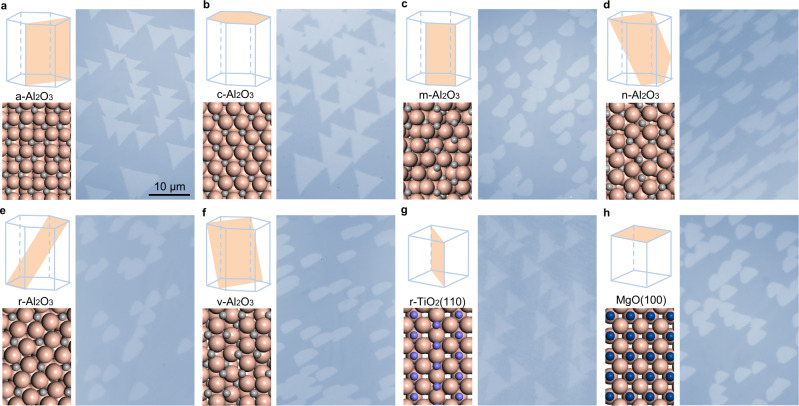
Universal growth of unidirectionally aligned MoS_2_ grains on various substrates. Schematic diagrams of lattice structures of the substrates and optical images of MoS_2_ grains grown on them. Unidirectionally aligned MoS_2_ grains can be produced on a- (**a**), c- (**b**), m- (**c**), n- (**d**), r- (**e**), v-Al_2_O_3_ (**f**), r-TiO_2_(110) (**g**), and MgO(100) surfaces (**h**). The orange, grey, violet and dark-blue spheres correspond to O, Al, Ti, and Mg atoms, respectively. The orange shaded planes in the lattice structures show the certain surface of the Al_2_O_3_, TiO_2_ and MgO substrates. The scale bars for (**a**–**h**) are the same.

In addition to the vicinal c-plane Al_2_O_3_ surface, we found that the current strategy for epitaxial growth of 2D single crystals is applicable for many other substrates. If the nucleation time matches the time of step edge formation well, unidirectionally aligned TMDs of different types can be easily grown on various substrates. As shown in Fig. 3a–h, the growth of unidirectionally aligned MoS_2_ grains was realized on various vicinal a, c, m, n, r, and v planes of Al_2_O_3_, the vicinal rutile TiO_2_(110) surface and the vicinal MgO(100) surface (Supplementary Figs. 10, 11). Once again, we noticed that the alignment of the MoS_2_ grains does not depend on the step edge direction (Supplementary Figs. 12, 13). Besides MoS_2_, we also realized the growth of unidirectionally aligned WS_2_, NbS_2_, MoSe_2_, WSe_2_ and NbSe_2_ grains (Supplementary Fig. 14). Thus, the epitaxial growth of non-centrosymmetric two-dimensional single-crystal metal dichalcogenides can be robustly achieved on many different substrates by initiating the TMDs growth during the step edge formation process.

### Mechanism of the simultaneous-formation-guided epitaxy

To deeply understand the mechanism for the universal epitaxy, we performed theoretical analysis with density functional theory (DFT) calculations. Our study clearly showed that (i) the step edge is essential for breaking the centrosymmetry of the substrate for the epitaxial growth of two-dimensional single-crystal metal dichalcogenides and (ii) the strong interaction between the 2D material and the immature step edges during the annealing process ensures the initial nucleation of 2D grains near the step edges to break the equivalence of the antiparallel grains subsequently for unidirectional alignment.

**Fig. 4:**
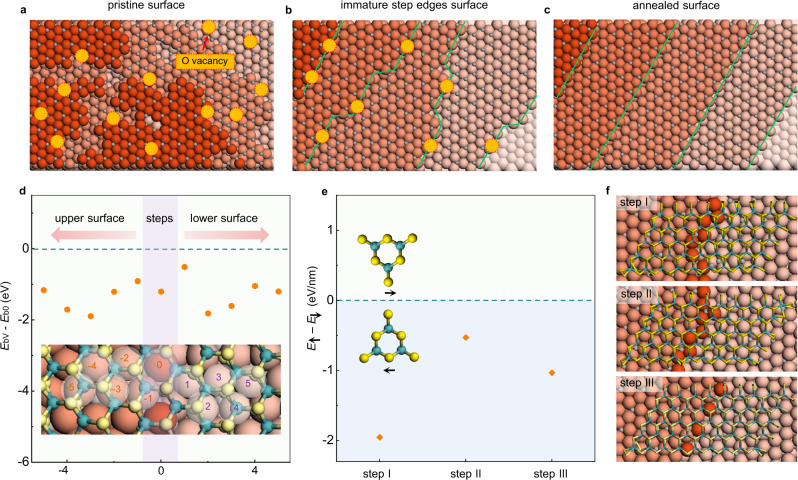
Mechanism of the simultaneous-formation-guided epitaxial growth of MoS_2_ on vicinal c-Al_2_O_3_. **a**–**c** Schematic diagrams of the evolution of the c-Al_2_O_3_ surface during annealing at high temperature. **a** The original c-Al_2_O_3_ surface is full of disorders and no clear separation of step edges and terraces. **b** During annealing, oxygen vacancies on terraces were gradually annealed, while some disorders, such as oxygen vacancies, still appeared in the blurred step edges. **c** After a long annealing time, the substrate is fully reconstructed with ultra-flat terraces separated by parallel step edges, and all the disorders on the substrate disappears. The red dotted circles represent the oxygen vacancy. The yellow, green, pink and grey spheres represent the S, Mo, O, and Al atoms, respectively. The green lines show the edges of the steps. **d** The binding energy difference of a MoS_2_ grain on a straight parallel step without O vacancy (*E*_b0_) and on a defective step with different O vacancy at different sites (*E*_bV_). The negative values of (*E*_bV_*–E*_b0_) reveals a stronger interaction between a MoS_2_ grain and the defective steps. Inset: the different positions of O vacancy used in our calculations are marked by numbers and semi-transparent circles. The horizontal dashed line corresponds to *E*_bV_ = *E*_b0_. The light purple and light green shaded areas correspond to the steps and terrace areas. **e** Energy difference between two antiparallel MoS_2_ grains that cross three different types of step edges (I, II and III). The significant energy differences (0.5–2.0 eV/nm) show that all these step edges are able to break the symmetry of antiparallel MoS_2_ grains on the c-Al_2_O_3_. All negative values imply that the alignment of the MoS_2_ grain is not sensitive to the direction of the step edge and uni-alignment MoS_2_ grains on various step edges can be formed. Inset: The positive and negative energy differences corresponding to the preferential of two antiparallelly aligned MoS_2_ grains. The horizontal dashed line corresponds to *E*_↑_=*E*_↓_. The light blue and light green shaded area correspond to *E*_↑_<*E*_↓_ and *E*_↑_>*E*_↓_, respectively. **f** The MoS_2_ grain on three types of step edges.

Taking MoS_2_ growth on a vicinal c-Al_2_O_3_ surface as an example, an as-cut Al_2_O_3_ surface is generally full of disorders, where the step edges and terraces are randomly distributed and cannot be distinguished (Fig. 4a). After long time annealing, the surface is fully reconstructed and ultra-flat terraces separated by parallel sharp step edges can be seen (Fig. 4c). During the reconstruction process, most of the disorders, such as oxygen vacancies, on terraces are annealed but those near step edges are not (Supplementary Fig. 15)^31–34^. These immature step edges are expected to have many defects and thus are chemically active to ensure that the nucleation of most 2D grains occurs near step edges (Fig. 4b). We calculated the binding energy of a MoS_2_ near an oxygen vacancy. The results clearly showed that an oxygen vacancy can enhance the binding of a MoS_2_ flake to the substrate by Δ*E*_b_ ~1.0 eV (Fig. 4d). We also compare the MoS_2_ cluster nucleation on sapphire surface without steps, with perfect step edges, and with defective step edges, respectively (Supplementary Fig. 16). The result shows that the defective step edge apparently results in a stronger binding with MoS_2_ compared to both flat terrace and perfect step edge. Especially, the binding energy continuous decreases with the increase of the number of O vacancies. Such a stronger binding leads to much easier TMD nucleation near the immature step edges. Therefore, the simultaneous formation of TMD nuclei and substrate steps guarantee most nucleation occurs around the step edges.

Next, let’s consider the mechanism of unidirectional alignment of nucleated TMDs near step edges. Because of the C_2_ symmetry of the c-Al_2_O_3_ substrate, the couplings between a MoS_2_ lattice and the c-Al_2_O_3_ terrace lead to two equivalent antiparallel alignments of MoS_2_^25^. However, once the nucleation started near the step edges of a substrate, the equivalency between the antiparallel TMDs lattices could be broken and ensured the unidirectional alignment of MoS_2_ grains (Fig. 4e, f). Our calculations clearly show that the symmetry of the antiparallel aligned MoS_2_ grains can be broken by step edges along different directions and the most stable alignment remains for a large variation in step edge directions. As shown in Fig. 4e, f, all three different step edges distinguish the antiparallel MoS_2_ by ~1 eV/nm, and the preferred alignment is the same. In principle, this mechanism has broad applications for the growth of a variety of 2D materials on desired substrates, and thus is universal for the growth of 2D single crystals (calculations of WS_2_ on c-Al_2_O_3_ and WS_2_/MoS_2_ on a-Al_2_O_3_ are shown in Supplementary Fig. 17).

## Discussion

Utilizing the full potential of 2D materials largely depends on the vertical integration of different single-crystal films^35^. At present, the transfer and integration techniques of 2D materials are very mature. However, the kinds of 2D single crystals that can be prepared on the wafer scale are very limited thus far. Our methodology opens an avenue to grow many kinds of 2D single crystals on desired substrates and will be very likely to proceed their integration for high-end electronic and optical applications.

## Methods

### Detailed information of c-Al_2_O_3_

Raw Material: 99,999%, High Purity, Monocrystalline Al_2_O_3_; Growth Method: Kyropoulos; Crystal Grade: Optical Grade 1; Processing Grade: Epi-ready; Diameter: 50.8 mm + /− 0.1 mm; Thickness: 430 μm + /− 25 μm; Primary Flat Orientation A-plane (11–20) +/− 0.2°; Primary Flat Length: 16.0 mm + /− 1.0 mm; Front Surface: Epi-polished, Ra < 0.2 nm; Back Surface: Fine ground, Ra = 0.8 μm–1.2 μm.

### Growth of single-crystal MoS_2_ monolayer on vicinal c-Al_2_O_3_

MoS_2_ monolayer grains and films were grown on unannealed Al_2_O_3_ substrates (a, c, m, n, r, and v planes, Dongda Times (Chengdu) Technology Co., LTD) in a chemical vapour deposition (CVD) system with three temperature zones. S (Alfa Aesar, 99.9%) powder, MoO_3_ (Alfa Aesar, 99.99%) powder and NaCl (Greagent, 99.95%) mixture, Al_2_O_3_ were placed on the upstream end of the quartz tube, temperature zone-I and temperature Zone-III of the tube furnace, respectively. During the growth process, under a mixed gas flow (Ar, 30 sccm; H_2_, 0–5 sccm), the temperature zone-I, II and III of the tube furnace were heated to 565, 850, and 975 °C within 50 min, respectively. During this process, the sapphire substrate would start construct and the immature steps started to appear. When zone-I was heated to 400 °C, the S (Alfa Aesar, 99.9%) powder was heated to 150 °C, within 15 min by a heating belt. When zone-I was heated to 510 °C, a little amount of oxygen was introduced. The growth time was 10–40 min to obtain MoS_2_ grains or films. After the growth, the system was naturally cooled to room temperature with 300 sccm Ar. The detailed growth setup and temperature ramps can be seen in Supplementary Fig. 18.

### Growth of WS_2_, NbS_2_, MoSe_2_, WSe_2_ and NbSe_2_ on vicinal c-Al_2_O_3_

The growth recipes were very similar to that for MoS_2_ except for the replacement of MoO_3_ by WO_3_ or Nb_2_O_5_ source, S by Se source and the temperature settings were adjusted accordingly. During WS_2_ growth, the temperature of the S source, zone-I, II, III, were set as 150, 645, 850, and 975 °C under gas flow (Ar, 30 sccm). During NbS_2_ growth, the temperatures were set as 150, 745, 850, and 965 °C. During MoSe_2_ growth, the temperatures were set as 250, 565, 850, and 975 °C. During WSe_2_ growth, the temperatures were set as 250, 645, 850, and 975 °C. During NbSe_2_ growth, the temperatures were set as 250, 745, 850, and 965 °C.

### Characterization

(i) LEED measurements were performed using Omicron LEED system in UHV with base pressure < 3 × 10^−7^ Pa. AFM measurements were performed using Bruker Dimensional ICON under atmospheric environment.
(ii) Optical measurements. Optical images were conducted with an Olympus microscope (Olympus BX51). Raman spectra were obtained with a home-made Raman system with laser excitation wavelength of 532 nm and power of ~0.5 mW. Low-temperature PL spectra were obtained at 15 K using a home-made optical cryostat with laser excitation wavelength of 532 nm and power of ~8 μW. Polarized light was generated with a super-achromatic quarter-wave plate (Thorlabs SAQWP05M-700) and the photoluminescence was analysed through the same quarter-wave plate and a linear polarizer. SHG mapping was obtained using the same system under excitation from a femtosecond laser centred at 820 nm with average power of 800 μW (Spectra-Physics Insight system with pulse duration of 100 fs and repetition rate of 80 MHz).
(iii) TEM characterization. The MoS_2_ sample were transferred onto homemade monolayer graphene TEM grids using the polymethyl-methacrylate-based transfer technique. Graphene TEM grids were made by transferring large-area monolayer single-crystal graphene on commercial holey carbon TEM grids (Zhongjingkeyi GIG-2010-3C). STEM experiments were performed in FEI Titan Themis G2 300 operated at 80 kV.
(iv) Device fabrications and measurements. The FETs were fabricated through standard microfabrication process by electron beam lithography techniques. The MoS_2_ sample was transferred from sapphire substrate by wet method assisted by KOH solution (1 Mol/L, 110 °C). The Bi/Au contact electrodes (~10/30 nm) were fabricated by e-beam deposition system with a low vacuum ~3 × 10^−7^ Pa. All the electrical measurements were carried out in a Janus probe station (base pressure 10^−6^ Torr) with Agilent semiconductor parameter analyser (B1500, high resolution modules) at room temperature.

### Computational details

Geometric optimization and energy calculations of the MoS_2_/c-Al_2_O_3_ systems were carried out using density functional theory (DFT) as implemented in Vienna Ab-initio Simulation Package. The generalized gradient approximation (GGA) with the Perdew–Burke–Ernzerhof (PBE) exchange-correlation function was used with the plane-wave cutoff energy set at 400 eV for all calculations. The dispersion-corrected DFT-D_3_ method was used because of its good description of long-range vdW interactions for multi-layered 2D materials. The geometries of the structures were relaxed until the force on each atom was less than 0.01 eV Å^−1^, and the energy convergence criterion of 1 × 10^−5^ eV was met. The Al_2_O_3_ surfaces were modelled by a periodic slab and some bottom layers were fixed to mimic the bulk, a 1 × 1 × 1 Monkhorst–Pack k-point mesh was adopted. The binding energies of the MoS_2_ – substrate hybrid, namely, *E*_b_ = (*E*_hyb_ – *E*_MoS2_ –*E*_sub_)/S, was calculated using the relaxed structures, where *E*_*hyb*_ is the total energy of the hybrid; *E*_MoS2_ and *E*_sub_ represent the energies of MoS_2_ and the substrate, respectively; and S is the area of the MoS_2_ cluster. To estimate the Al_2_O_3_ step–MoS_2_ interaction, two antiparallel MoS_2_ nanoribbons with a length of about 1.6 nm were placed on the steps of the c-Al_2_O_3_ surface and then relaxed. The energy difference was defined as *ΔE* = *E*_*↑*_ – *E*_*↓*_, *E*_*↑*_ = *E*_1_/L and *E*_*↓*_ = *E*_2_/L, where *E*_1_ and *E*_2_ are the total energies of the hybrid system, respectively, and L is the length of the nanoribbon. Similar calculations were also conducted for the MoS_2_/a-Al_2_O_3_ systems.

## Supplementary information

supplementary information

## Data Availability

The authors declare that the data supporting the findings of this study are available within the paper, Supplementary Information and Source Data. Extra data are available from the corresponding authors upon request. Source data are provided with this paper.

## Source data

Source Data

## References

[1] Dumcenco D (2015). Large-area epitaxial monolayer MoS_2_. ACS Nano.

[2] Gronborg SS (2015). Synthesis of epitaxial single-layer MoS_2_ on Au(111). Langmuir.

[3] Song XJ (2015). Chemical vapor deposition growth of large-scale hexagonal boron nitride with controllable orientation. Nano Res..

[4] Yin J (2015). Aligned growth of hexagonal boron nitride monolayer on germanium. Small.

[5] Tay RY (2016). Synthesis of aligned symmetrical multifaceted monolayer hexagonal boron nitride single crystals on resolidified copper. Nanoscale.

[6] Meng JH (2017). Aligned growth of millimeter-size hexagonal boron nitride single-crystal domains on epitaxial nickel thin film. Small.

[7] Uchida Y, Iwaizako T, Mizuno S, Tsuji M, Ago H (2017). Epitaxial chemical vapour deposition growth of monolayer hexagonal boron nitride on a Cu(111)/sapphire substrate. Phys. Chem. Chem. Phys..

[8] Aljarb A (2017). Substrate lattice-guided seed formation controls the orientation of 2D transition-metal dichalcogenides. ACS Nano.

[9] Yu H (2017). Wafer-scale growth and transfer of highly-oriented monolayer MoS_2_ continuous films. ACS Nano.

[10] Taslim AB (2019). Synthesis of sub-millimeter single-crystal grains of aligned hexagonal boron nitride on an epitaxial Ni film. Nanoscale.

[11] Li N (2020). Large-scale flexible and transparent electronics based on monolayer molybdenum disulfide field-effect transistors. Nat. Electron..

[12] Wang QQ (2020). Wafer-scale highly oriented monolayer MoS_2_ with large domain sizes. Nano Lett..

[13] Pan SY (2021). Effect of substrate symmetry on the orientations of MoS_2_ monolayers. Nanotechnology.

[14] Tumino F (2021). Hydrophilic character of single-layer MoS_2_ grown on Ag(111). J. Phys. Chem. C..

[15] Chen L (2015). Step-edge-guided nucleation and growth of Aligned WSe_2_ on sapphire via a layer-over-layer growth mode. ACS Nano.

[16] Li JD (2016). Growth of polar hexagonal boron nitride monolayer on nonpolar copper with unique orientation. Small.

[17] Li SS (2018). Vapour-liquid-solid growth of monolayer MoS_2_ nanoribbons. Nat. Mater..

[18] Chen TA (2020). Wafer-scale single-crystal hexagonal boron nitride monolayers on Cu (111). Nature.

[19] Aljarb A (2020). Ledge-directed epitaxy of continuously self-aligned single-crystalline nanoribbons of transition metal dichalcogenides. Nat. Mater..

[20] Ma ZP (2020). Epitaxial growth of rectangle shape MoS_2_ with highly aligned orientation on twofold symmetry a-plane sapphire. Small.

[21] Yang PF (2020). Epitaxial growth of centimeter-scale single-crystal MoS_2_ monolayer on Au(111). ACS Nano.

[22] Choi SH (2021). Epitaxial single-crystal growth of transition metal dichalcogenide monolayers via the atomic sawtooth Au surface. Adv. Mater..

[23] Chubarov M (2021). Wafer-scale epitaxial growth of unidirectional WS_2_ monolayers on sapphire. ACS Nano.

[24] Li J (2021). Single-Crystal MoS_2_ Monolayer Wafer Grown on Au (111) Film Substrates. Small.

[25] Li TT (2021). Epitaxial growth of wafer-scale molybdenum disulfide semiconductor single crystals on sapphire. Nat. Nanotechnol..

[26] Wang L (2019). Epitaxial growth of a 100-square-centimetre single-crystal hexagonal boron nitride monolayer on copper. Nature.

[27] Wang JH (2022). Dual-coupling-guided epitaxial growth of wafer-scale single-crystal WS_2_ monolayer on vicinal a-plane sapphire. Nat. Nanotechnol..

[28] Xu XZ (2017). Ultrafast epitaxial growth of metre-sized single-crystal graphene on industrial Cu foil. Sci. Bull..

[29] Cao T (2012). Valley-selective circular dichroism of monolayer molybdenum disulphide. Nat. Commun..

[30] Radisavljevic B, Radenovic A, Brivio J, Giacometti V, Kis A (2011). Single-layer MoS_2_ transistors. Nat. Nanotechnol..

[31] Benabid F, Notcutt M, Loriette V, Ju L, Blair DG (2000). X-ray induced absorption of high-purity sapphire and investigation of the origin of the residual absorption at 1064 nm. J. Phys. D. Appl. Phys..

[32] Lai CC (2016). Ligand-driven and full-color-tunable fiber source: Toward next-generation clinic fiber-endoscope tomography with cellular resolution. ACS Omega.

[33] Lushchik A (2021). Evidence for the formation of two types of oxygen interstitials in neutron-irradiated alpha-Al_2_O_3_ single crystals. Sci. Rep..

[34] Zhang MF (2011). Effects of neutron irradiation and subsequent annealing on the optical characteristics of sapphire. Phys. B.

[35] Liu Y (2016). Van der Waals heterostructures and devices. Nat. Rev. Mater..

