# Supplementary material for: Universal epitaxy of non-centrosymmetric two-dimensional single-crystal metal dichalcogenides
Source: Nat Commun. 2023 Feb 3;14:592. doi: 10.1038/s41467-023-36286-6 (PMC9898269; doi:10.1038/s41467-023-36286-6)
Supplement: Supplementary file 1 — supplementary information [file 41467_2023_36286_MOESM1_ESM.pdf]

Supplementary information for

Universal epitaxy of non-centrosymmetric two-dimensional single-crystal metal dichalcogenides

Peiming Zheng[#], Wenya Wei[#], Zhihua Liang[#], Biao Qin[#], Jinpeng Tian, Jinhuan Wang, Ruixi Qiao, Yunlong Ren, Junting Chen, Chen Huang, Xu Zhou, Guangyu Zhang, Zhilie Tang, Dapeng Yu, Feng Ding*, Kaihui Liu*, and Xiaozhi Xu*

The supplementary information includes:

Supplementary Tables 1-3

Supplementary Fig. 1-18

Supplementary Table 1 | Inconsistent growth behaviours of TMDs on substrates with steps					
**Reference**	**substrate**	**steps**	**TMDs**	**alignment**	**Symmetry breaking**
Nature Nanotechnology 2022, 17, 33	a-Al_2O_3	with steps	WS_2	99%	√
Small 2020, 16, 2000596	a-Al_2O_3	with steps	MoS_2	86%	√
Nature Nanotechnology 2021, 16, 1201	c-Al_2O_3	with steps	MoS_2	99%	√
ACS Nano 2021, 15, 2532	c-Al_2O_3	with steps	WS_2	>90%	√
ACS Nano 2015, 9, 8368	c-Al_2O_3	with steps	WSe_2	92%	√
Advanced Materials 2021, 33, 2006601	Au (533)	with steps	WS_2	>90%	√
Small 2021, 17, 2100743	Au(111)	with steps	MoS_2	99%	√
ACS Nano 2020, 14, 5036	Au (111)	with steps	MoS_2	98%	√
Nature Materials 2020, 19, 1300	β-Ga_2O_3	with steps	MoS_2	>90%	√
Nature Electronics 2020, 3, 711	c-Al_2O_3	with steps	MoS_2	50%	×
NANO Letters 2020, 20, 7193	c-Al_2O_3	with steps	MoS_2	50%	×
ACS Nano 2017, 11, 9215	c-Al_2O_3	with steps	MoS_2	60%	×
ACS Nano 2017, 11, 12001	c-Al_2O_3	with steps	MoS_2	50%	×
ACS Nano 2015, 9, 4611	c-Al_2O_3	with steps	MoS_2	56%	×
Langmuir 2015, 31, 35, 9700	Au(111)	with steps	MoS_2	50%	×
Nanotechnology 2021, 32, 095601	Au(111)	with steps	MoS_2	50%	×
J. Phys. Chem. C 2021, 125, 9479	Au(111)	with steps	MoS_2	50%	×

Supplementary Table 2 | Inconsistent growth behaviours of h-BN on substrates with steps				
**Reference**	**substrate**	**steps**	**alignment**	**Symmetry breaking**
Nature 2019, 570, 91	Cu(110)	with steps	99%	√
Nature 2020, 579, 219	Cu(111)	with steps	99%	√
Small 2016, 12, 3645	Cu(102)<br>Cu(103)	with steps	97%	√
Nanoscale, 2016,8, 2434	Cu(110)	with steps	50%	×
Nano Research 2015, 8, 3164	Cu(110)<br>Cu(111)	with steps	21%<br>54%	×
Phys. Chem. Chem. Phys., 2017,19, 8230	Cu(111)	with steps	50%	×
Small 2017, 13, 1604179	Ni(111)	with steps	50%	×
Nanoscale, 2019,11, 14668	Ni(111)	with steps	54%	×
Small 2015, 11, 5375	Ge (110)	with steps	52%	×

Supplementary Table 3 | Growth of TMDs under various conditions on annealed and un-annealed substrates

	Different growth temperature on c-Al_2O_3		Different sapphire planes					
	975 ℃	1000 ℃	a-Al_2O_3	c-Al_2O_3	m-Al_2O_3	n-Al_2O_3	r-Al_2O_3	v-Al_2O_3
immature step-edge	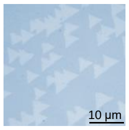	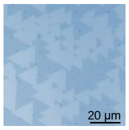	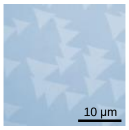	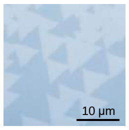	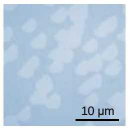	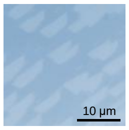	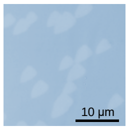	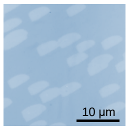
straight step-edge	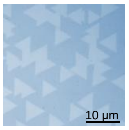	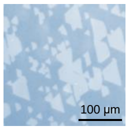	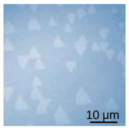	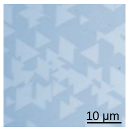	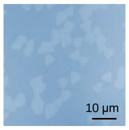	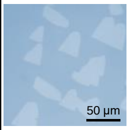	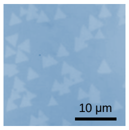	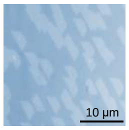
	different growth pressure on c-Al_2O_3		Different substrate types		different TMD materials			
	150 Pa	1 atm	r-TiO_2(110)	MgO(100)	WS_2	NbS_2	$MoSe_2$	WSe_2
immature step-edge	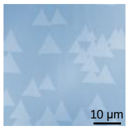	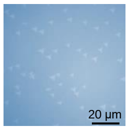	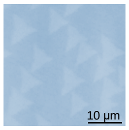	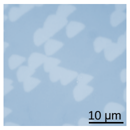	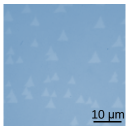	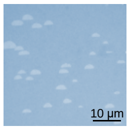	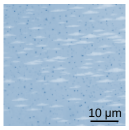	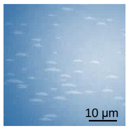
straight step-edge	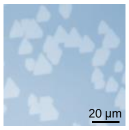	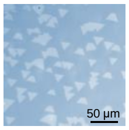	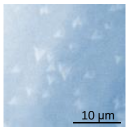	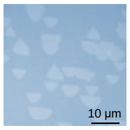	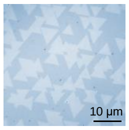	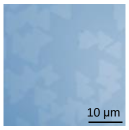	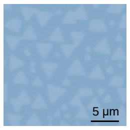	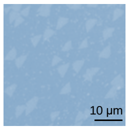

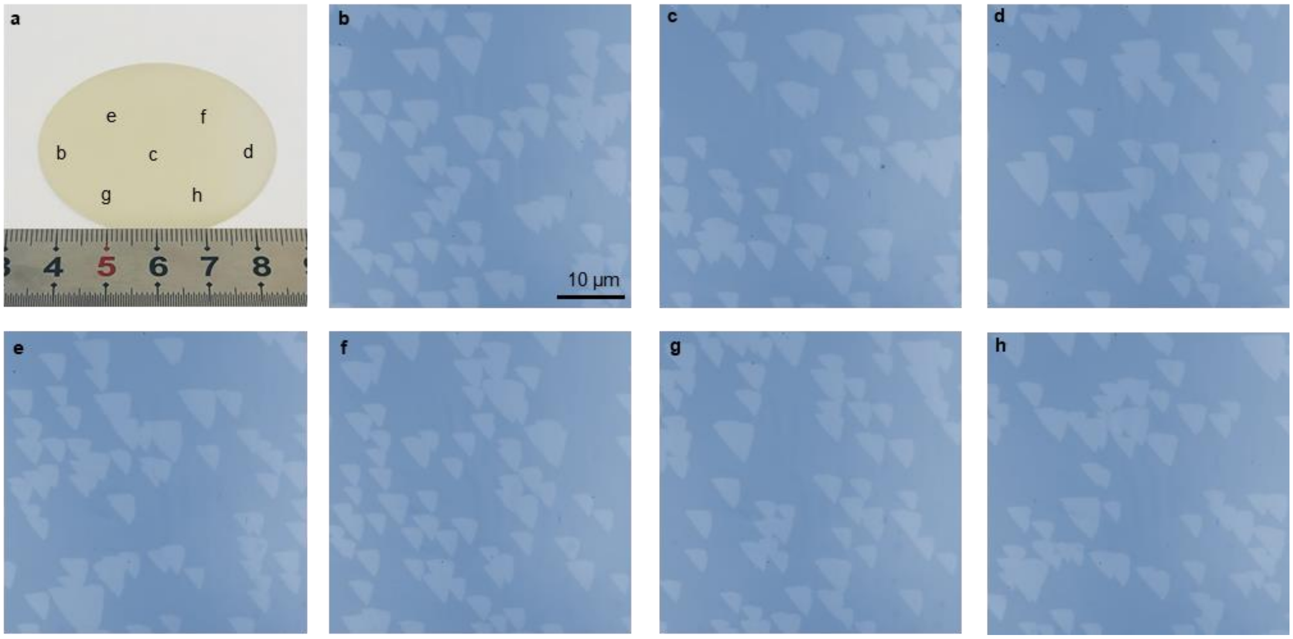

Supplementary Fig. 1 | Optical images of MoS_2 grains on vicinal c-Al_2O_3 wafer. a, Photograph of a 2-inch MoS_2 sample. **b-h**, Optical images of MoS_2 grains at different positions marked in **(a)**. The optical images at different positions show unidirectional orientation of the MoS_2 grains. The scale bars for **(b-h)** are the same.

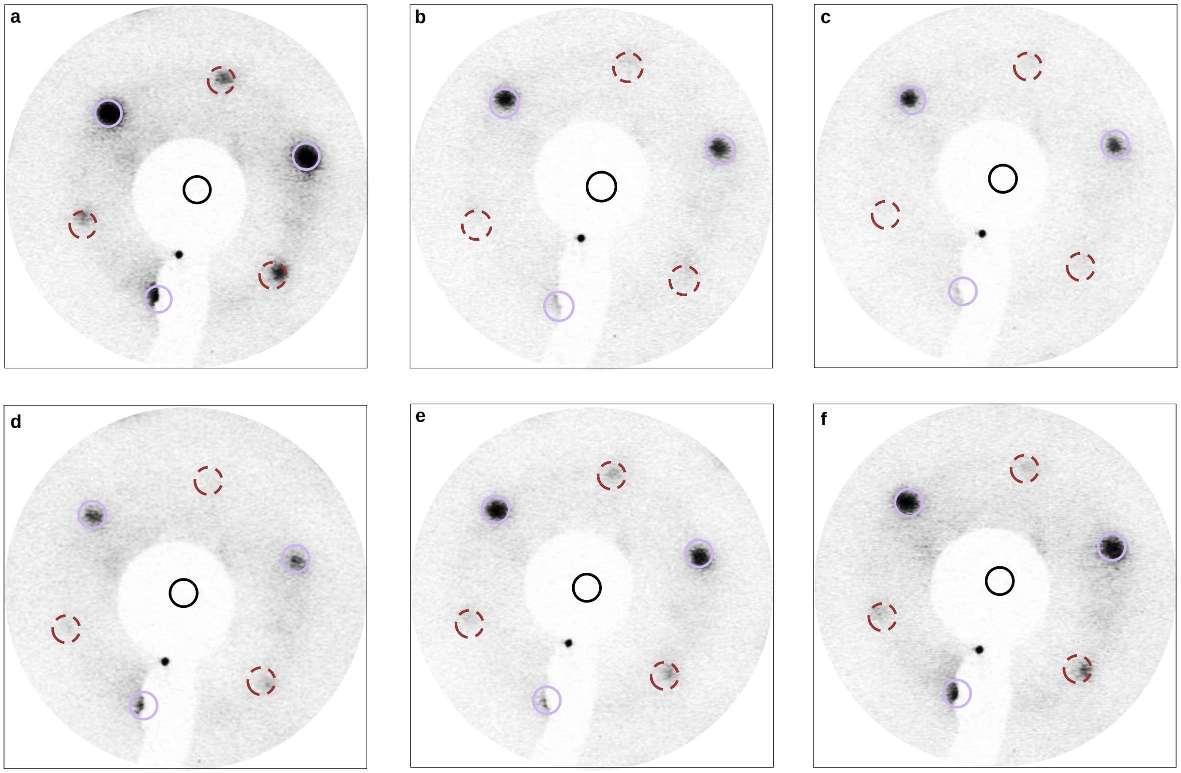

Supplementary Fig. 2 | LEED characterization of as-grown MoS_2 sample. a-f, LEED patterns of MoS_2 at different positions. The patterns at the different positions show two sets of triangular diffraction spots but with the same orientation, which proves the unidirectional alignment of the MoS_2 film.

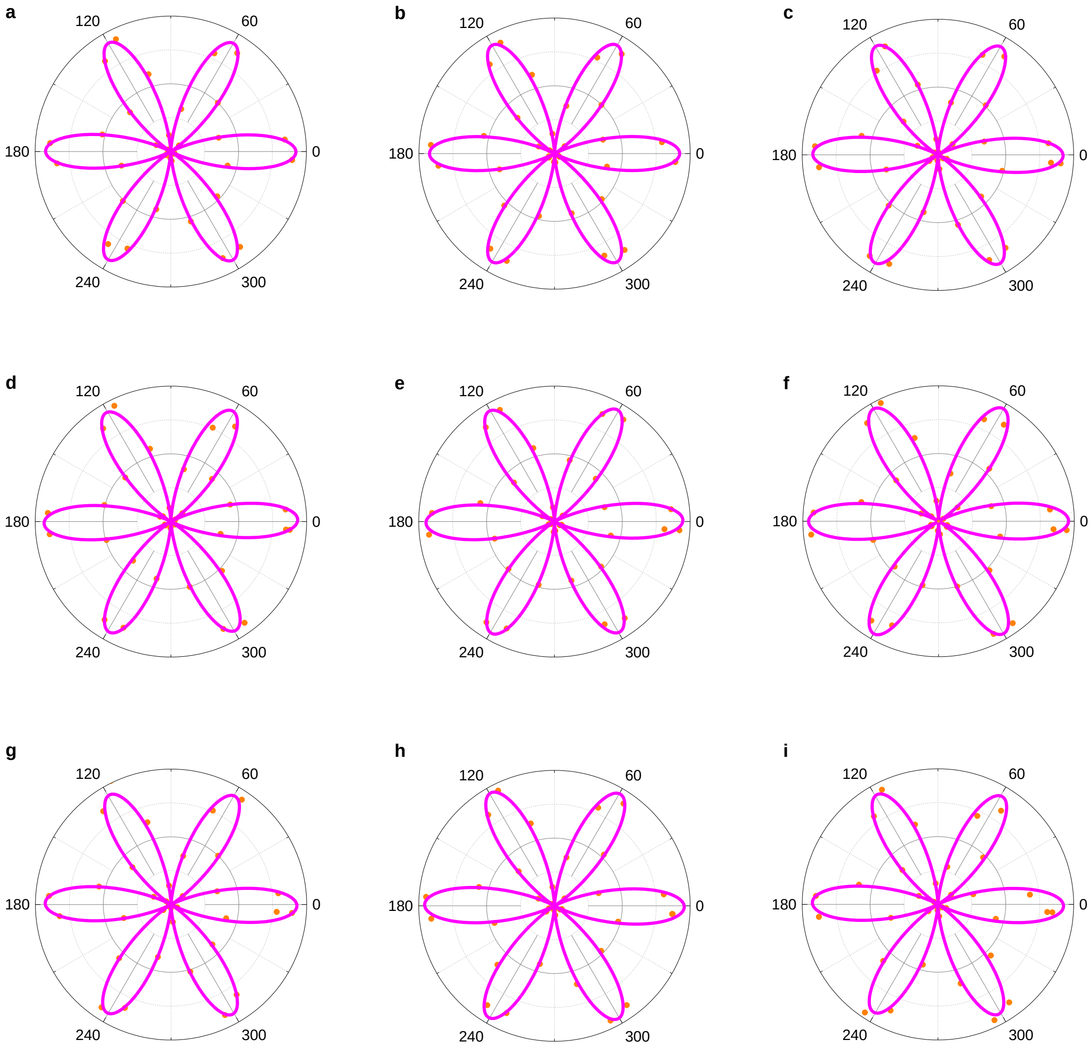

Supplementary Fig. 3 | Representative linearly polarized SHG patterns of MoS_2 at different positions. The nearly identical orientations of the patterns in **(a-i)** are consistent with the unidirectionally aligned MoS_2 grains.

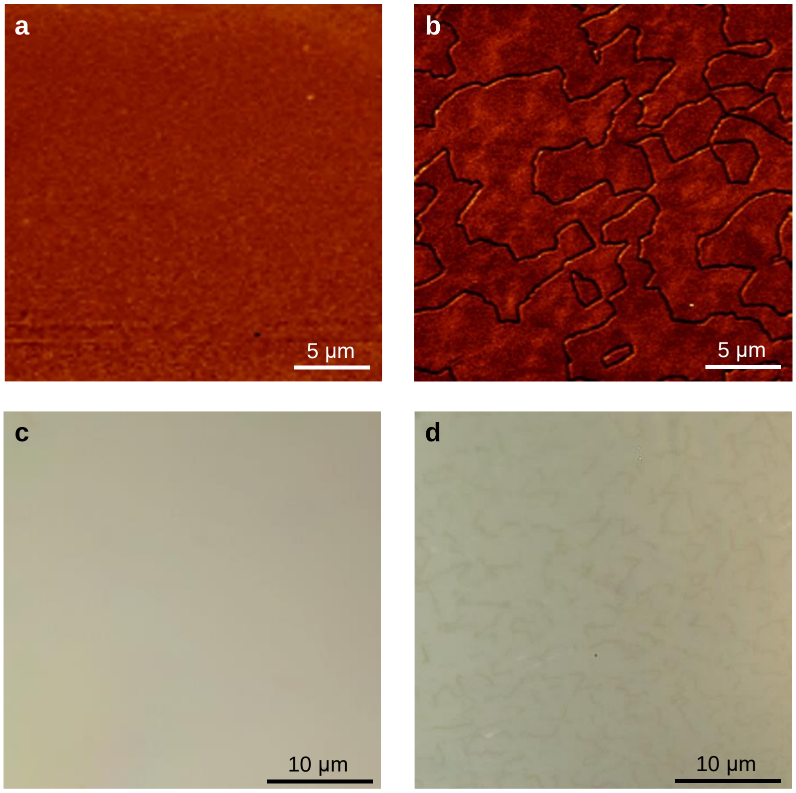

Supplementary Fig. 4 | Seamless stitching of aligned MoS_2 grains. a, SHG intensity mapping of unidirectionally aligned MoS_2 grains, the absence of any dark lines demonstrates the seamless stitching. **b,** SHG intensity mapping of anti-aligned MoS_2 gains. The dark line corresponds to the grain boundary. **c-d,** Optical images of MoS_2 films grown by the simultaneous-formation-guided mechanism growth **(c)** and ordinary growth **(d)** methods after hot H_2O vapour etching. The absence of any dark lines in MoS_2 film demonstrates the single-crystal nature of the MoS_2 films.

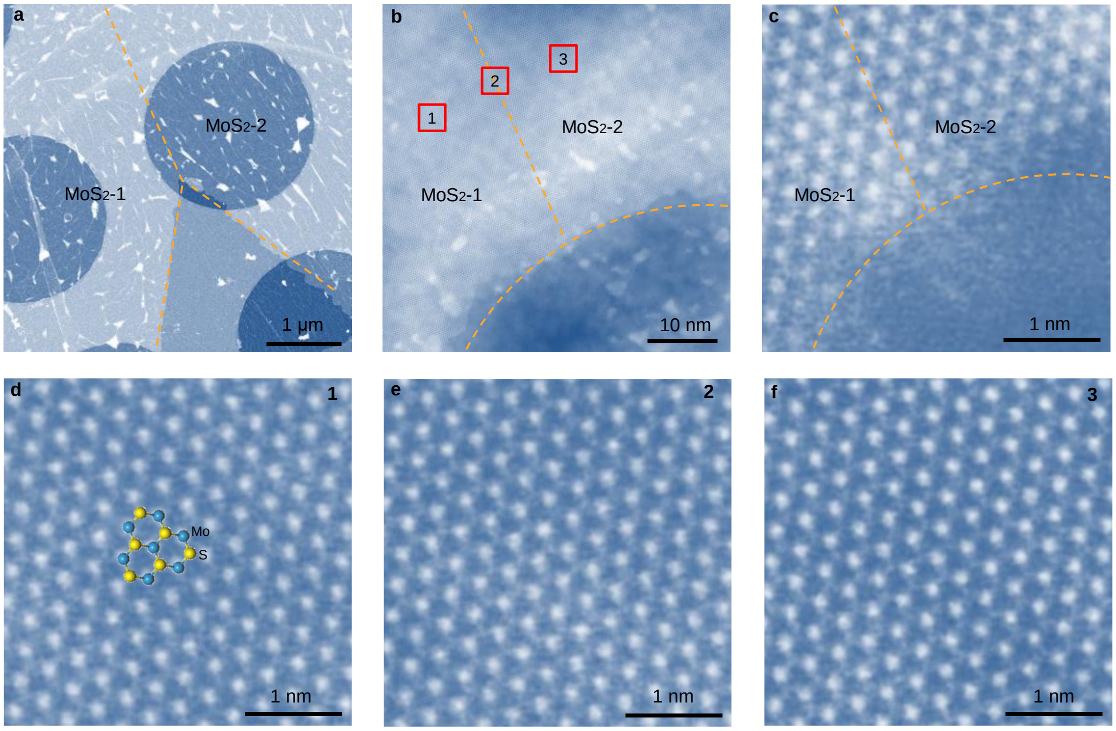

Supplementary Fig. 5 | STEM images of MoS_2 sample at the merged area. a-b, TEM images of merged MoS_2 grains at large scale. **c,** Zoom-in images of the merged areas shown in **(b)**. **d-f,** Zoom-in images of the merged areas at different positions marked in **(b)**.

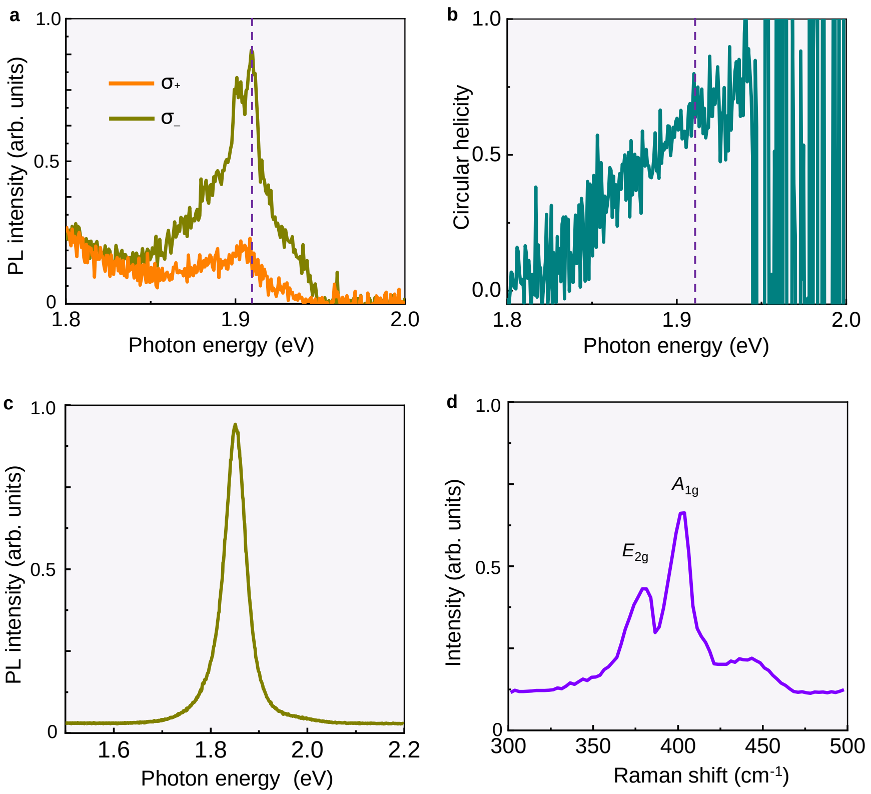

Supplementary Fig. 6 | Optical spectroscopic characterizations of MoS_2 monolayer grown on vicinal c-Al_2O_3. a, Circularly polarized PL spectra of MoS_2. **b,** Circular helicity calculated from the PL spectra in **(a).** A high circular helicity (up to 80%) was observed. **c-d,** Typical PL spectrum **(c)** and Raman spectrum **(d)** of MoS_2 sample at room temperature.

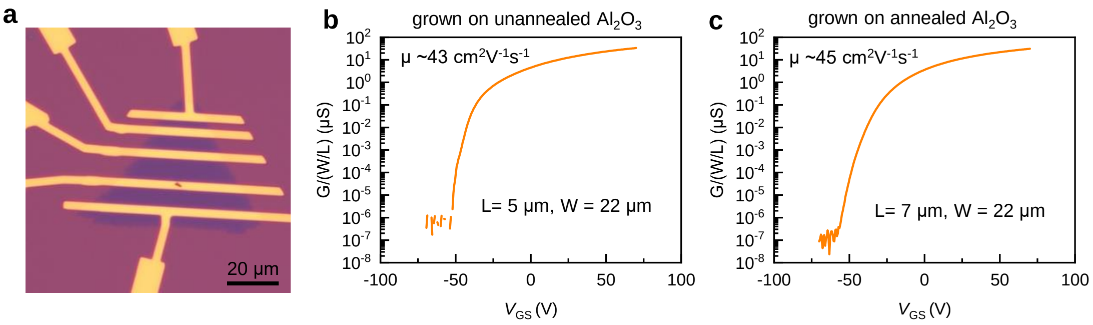

Supplementary Fig. 7 | Electrical measurements of as-grown MoS_2 samples. a, A tipical optical image of the FET device. **b-c,** Electrical measurement of an as-grown MoS_2 sample on unannealed **(b)** and annealed **(c)** Al_2O_3.

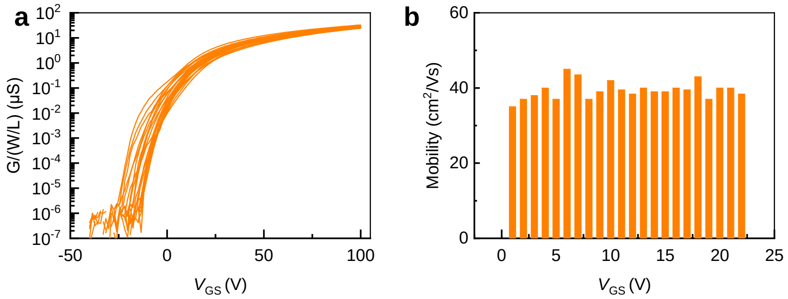

Supplementary Fig. 8 | Electrical measurements of MoS_2 sample. a, Transfer curves of 24 MoS_2 FETs devices fabricated at different locations of the 2-inch MoS_2 wafer. **b,** Statistical mobility of the 24 MoS_2 FET devices.

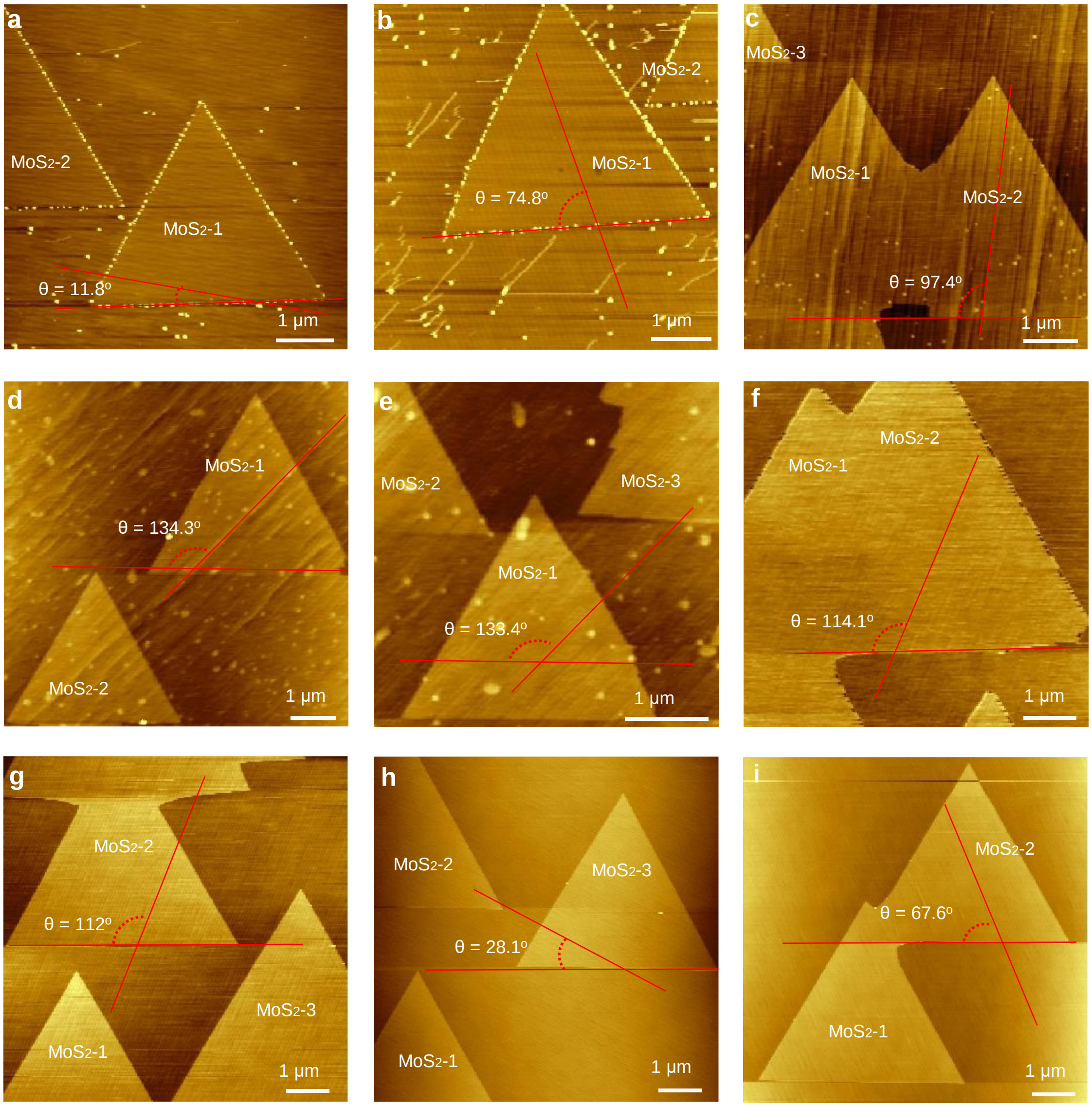

Supplementary Fig. 9 | AFM images of aligned MoS_2 islands on c-Al_2O_3 with different step orientations. The representative 9 different step orientations are shown in **(a-i)**.

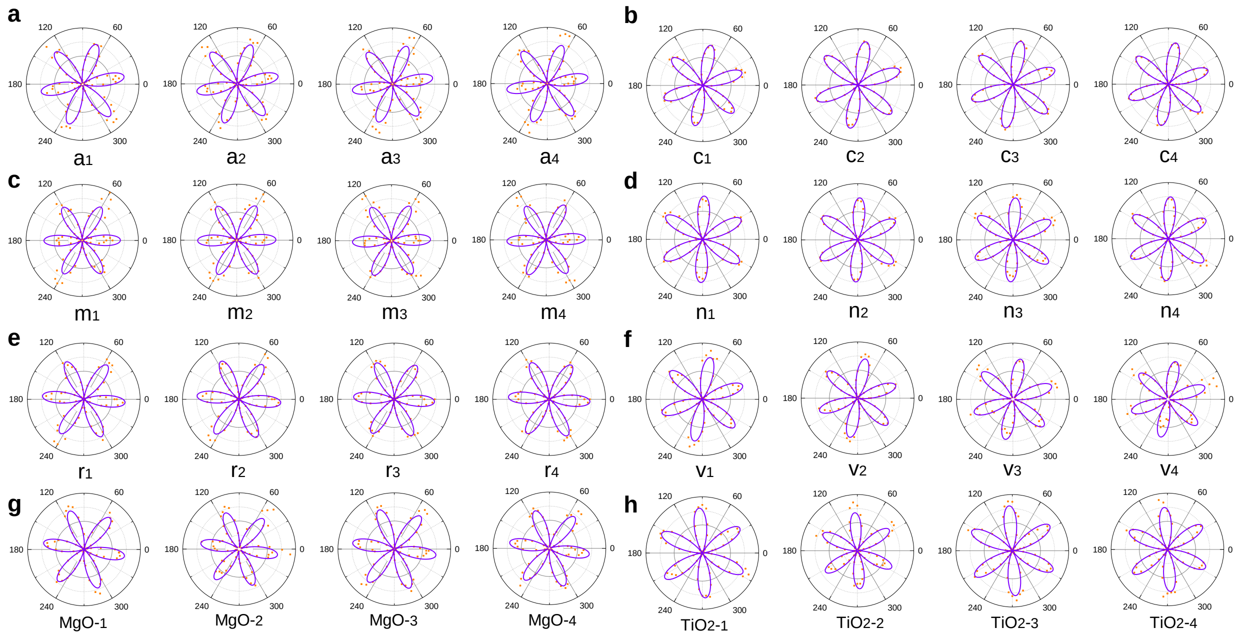

Supplementary Fig. 10 | The representative linearly polarized SHG six petal patterns at different positions of MoS_2 on various substrates. The SHG patterns of MoS_2 grown on a- **(a)**, c- **(b)**, m- **(c)**, n- **(d)**, r- **(e)** and v-plane sapphire **(f)** as well as MgO **(g)** and TiO_2 **(h)** substrates all show identical orientations, indicating the aligned lattice.

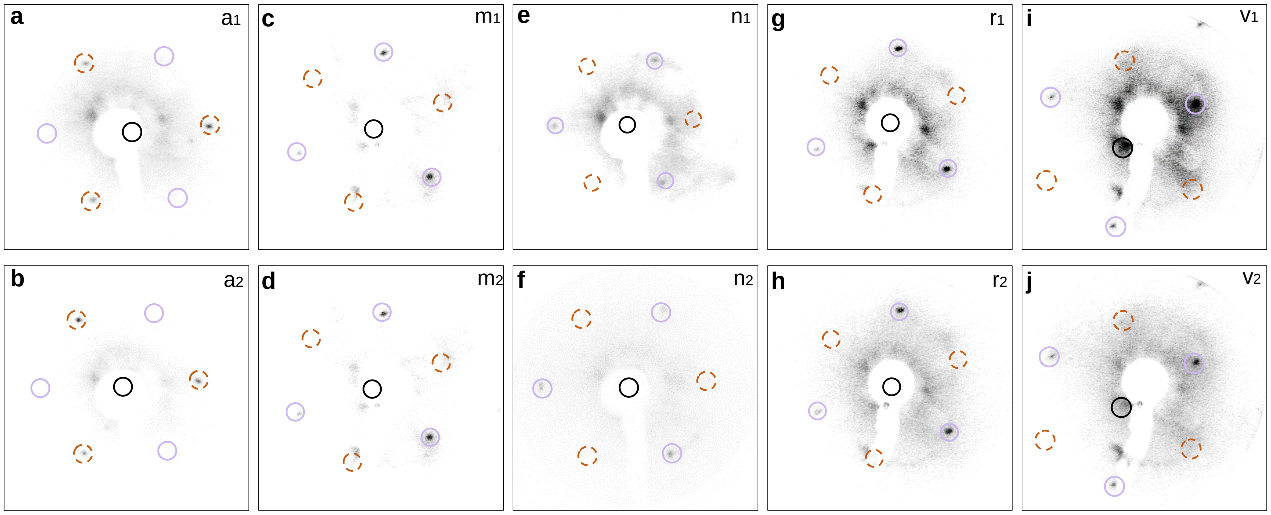

Supplementary Fig. 11 | LEED patterns of MoS_2 samples on various sapphire substrates. The patterns of MoS_2 grown on a- **(a-b)**, m- **(c-d)**, n- **(e-f)**, r- **(g-h)** and v-palne sapphire **(i-j)** all show identical orientations, indicating the single-crystal nature.

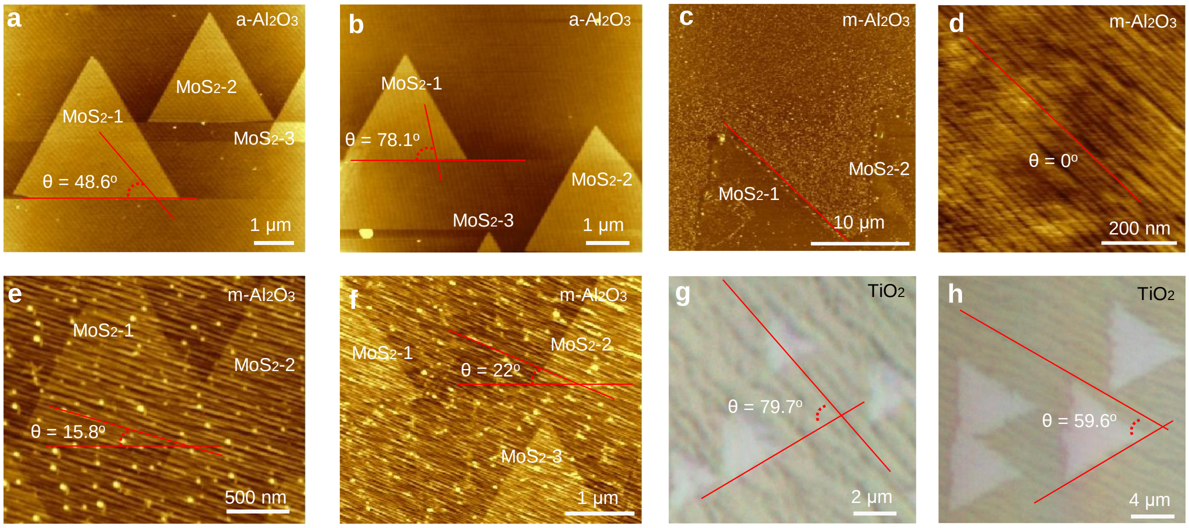

Supplementary Fig. 12 | AFM images of aligned MoS_2 islands on different substrates. a-b, AFM images of aligned MoS_2 islands on a-Al_2O_3**. c-f,** AFM images of aligned MoS_2 islands on m-Al_2O_3**. g-h,** Optical images of aligned MoS_2 islands on TiO_2.

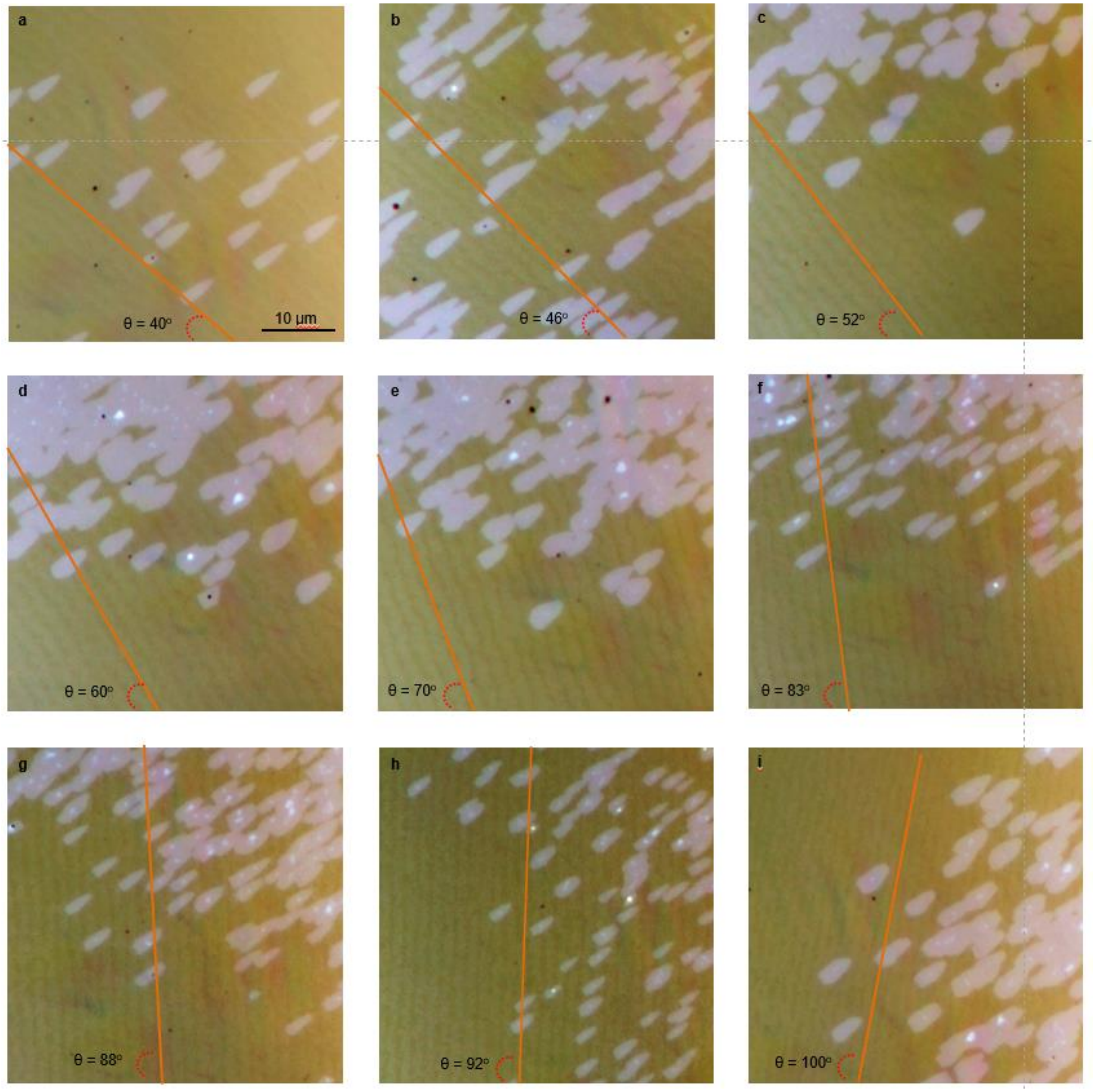

Supplementary Fig. 13 | Optical images of aligned MoS_2 islands on r-Al_2O_3. The image size of **(a-i)** are all the same.

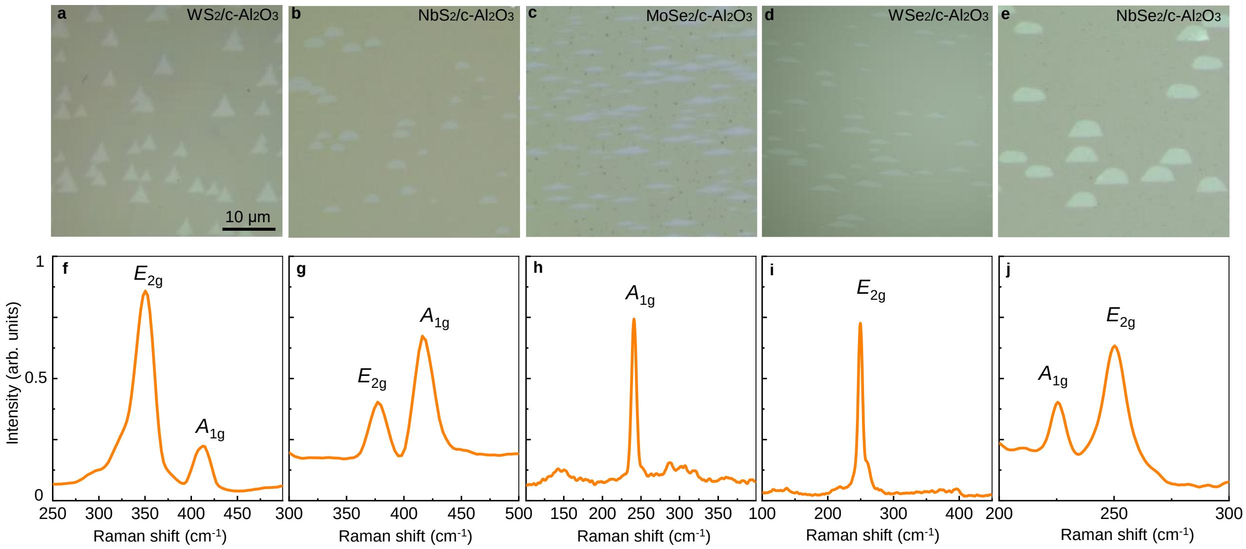

Supplementary Fig. 14 | Growth of unidirectional aligned WS_2, NbS_2, $MoSe_2$, WSe_2 and $NbSe_2$ grains on vicinal c-Al_2O_3. a-e, Optical images of the unidirectional aligned WS_2, NbS_2, $MoSe_2$, WSe_2 and $NbSe_2$ grains grown on c-Al_2O_3. The scale bars are the same. **f-j,** Typical Raman spectra of WS_2, NbS_2, $MoSe_2$, WSe_2 and $NbSe_2$ samples.

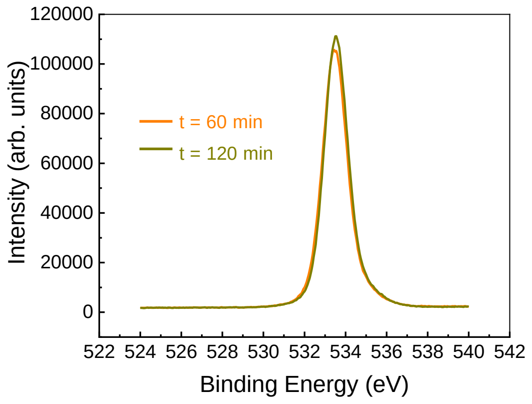

Supplementary Fig. 15 | XPS spectra of oxygen corresponding to the immature (t = 60 min) and mature steps (t = 120 min).

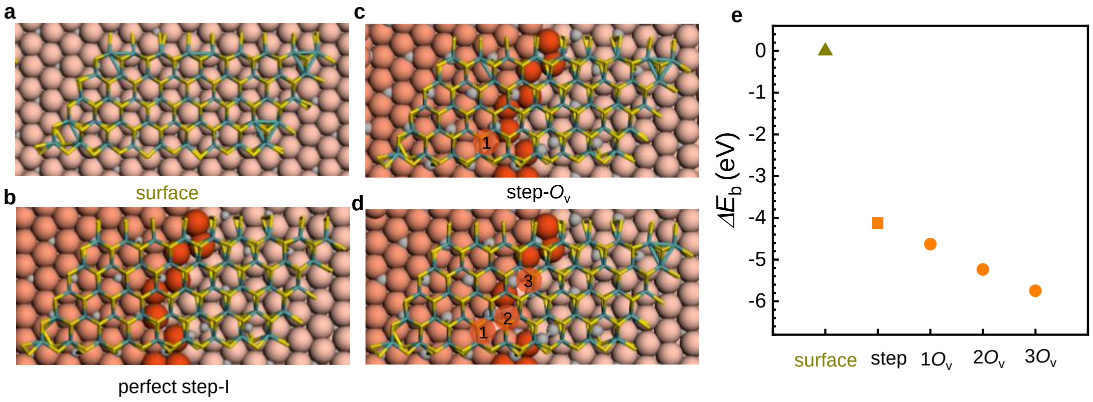

Supplementary Fig. 16 | Thermodynamic analysis of MoS_2 nucleation on sapphire surface without steps **(a)**, perfect step edges **(b)** and defective step edges **(c-d)**, respectively. Different positions of O vacancies are marked by black numbers and semi-transparent circles. **e,** The binding energy difference (ΔE_b) clearly shows that the defective steps are more active sites for MoS_2 nucleation compared to both flat surface and perfect steps. The defective steps are more active with the number of O vacancies increased.

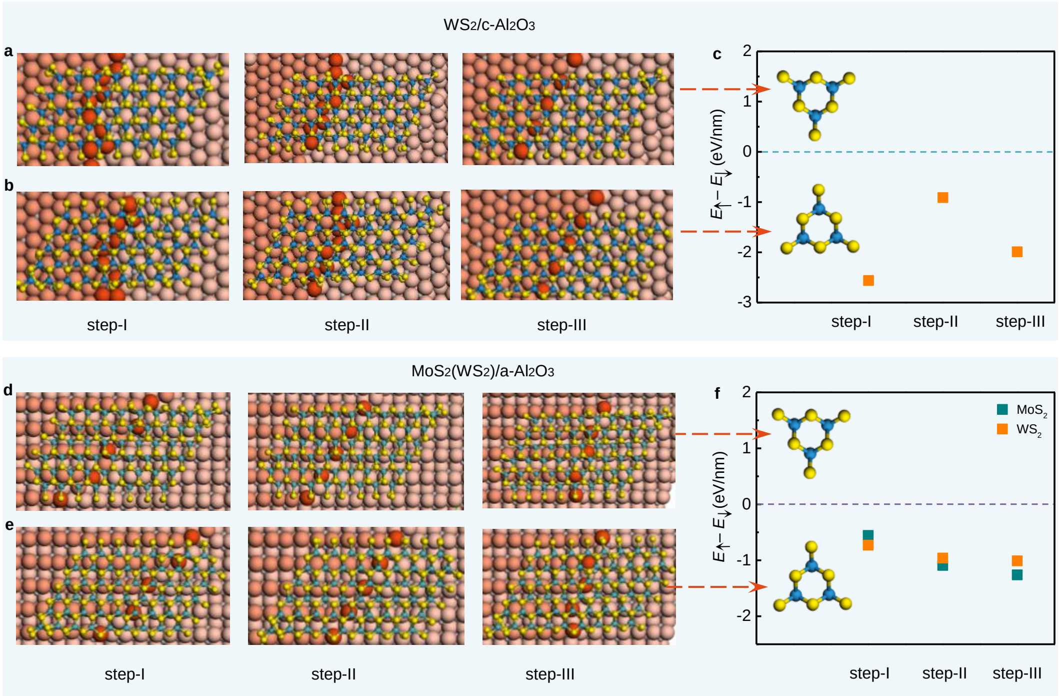

Supplementary Fig. 17 | Energy difference between two antiparallel WS_2 (MoS_2) grains that cross a step edge of vicinal a- or c-Al_2O_3. a-b, Schematic diagrams of two antiparallel WS_2 grains that across a step edge of c-Al_2O_3. **c,** Energy difference between two antiparallel WS_2 grains. **d-e,** Schematic diagrams of two antiparallel WS_2 (MoS_2) grains that across a step edge of a-Al_2O_3. **f,** Energy difference between two antiparallel WS_2 (MoS_2) grains. Steps along different directions all work for the breaking of the antiparallel alignments.

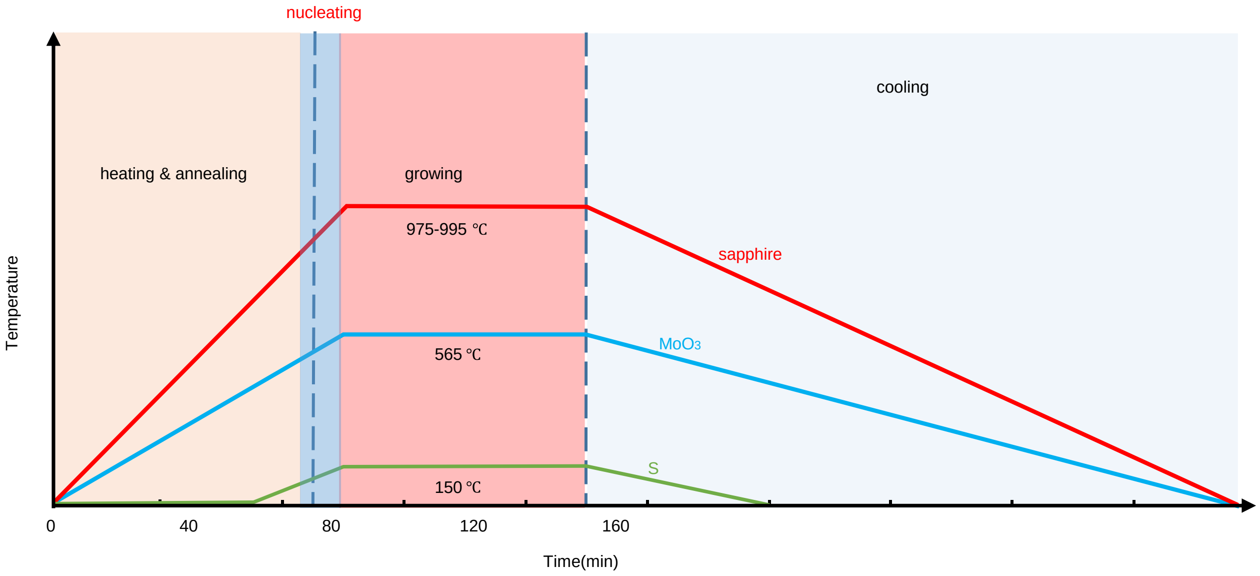

Supplementary Fig. 18 | Schematic diagrams of the growth setup and temperature ramps. The red, blue and green lines correspond to the temperature evolution of the sapphire substrate, MoO_3 and S, respectively. The orange, blue, pink and light blue spheres represent the heating/annealing, nucleating, growing, and cooling process, respectively.
